# Effect of Silicon Carbide Fiber Length on the Flexural Strength and Flexural Modulus of Short Silicon Carbide Fiber-Reinforced Resin

**DOI:** 10.3390/jfb15020030

**Published:** 2024-01-26

**Authors:** Norimasa Taka, Yujin Aoyagi, Keito Miida, Mitsugu Kanatani, Hiroshi Ogawa

**Affiliations:** 1 Division of Preventive Dentistry, Faculty of Dentistry, Graduate School of Medical and Dental Sciences, Niigata University, 2-5274 Gakkocho-dori, Chuo-ku, Niigata 951-8514, Japan; taka@dent.niigata-u.ac.jp; 2 Division of Bio-Prosthodontics, Faculty of Dentistry, Graduate School of Medical and Dental Sciences, Niigata University, 2-5274 Gakkocho-dori, Chuo-ku, Niigata 951-8514, Japan; aoyagiy@dent.niigata-u.ac.jp (Y.A.); kmiida@dent.niigata-u.ac.jp (K.M.); 3 Division of Biomimetics, Faculty of Dentistry, Graduate School of Medical and Dental Sciences, Niigata University, 2-5274 Gakkocho-dori, Chuo-ku, Niigata 951-8514, Japan; kanatani@dent.niigata-u.ac.jp

**Keywords:** silicon carbide fiber, short fiber-reinforced composite, flexural properties, dental composites

## Abstract

Silicon carbide fibers have superior flexural properties and chemical stability compared to glass fibers. We investigated the flexural strength and modulus of an experimental, short silicon carbide fiber-reinforced resin. Short silicon carbide fibers with lengths of ~0.5, 1, 2, and 3 mm were prepared and silanized. Urethane dimethacrylate and triethylene glycol dimethacrylate were mixed at a 70:30 wt% ratio and used as the matrix resins. Each length of short silicon carbide fibers and the matrix resin were combined using a mixing machine and then used for specimen preparation. The three-point bending test conditions were in accordance with ISO 4049:2009. The fracture surfaces of the specimens after the three-point bending test were observed using secondary electron images. The data were statistically analyzed with a one-way analysis of variance and Tukey’s HSD test (α = 0.05). The flexural strength and modulus of the specimens containing 2 mm or 3 mm silicon carbide fibers were significantly higher than the other specimens. The river pattern was observed more clearly in specimens containing shorter silicon carbide fibers, although this pattern was observed in all specimens.

## 1. Introduction

Composite resins are relatively inexpensive compared to ceramic materials and are widely used because of their superior esthetic properties compared to dental alloys [1,2,3,4]. In Japan, crown restorations using CAD/CAM resin blocks, produced by polymerizing composite resin under high pressure [5,6], abutment construction with fiber posts, and the creation of fixed partial prostheses called “high-strength hard resin bridges” using glass-fiber-reinforced resin are gaining increasing popularity. However, the mechanical strength of CAD/CAM resin blocks remains insufficient, and they are used only for single crowns or inlays. In contrast, the prosthodontic treatment for missing teeth using “high-strength hard resin bridges”, made of glass-fiber-reinforced resin, is covered by the national health insurance in Japan. However, it is limited to cases where the mandibular first premolars and mandibular first molars serve as the abutment teeth for missing mandibular second premolars.

The advantages of glass fibers include: (1) excellent esthetic properties owing to the use of a crown-colored matrix resin, made possible by the transparency of the fibers; (2) the efficient reinforcement effect of chemical bonding with the matrix resin; and (3) the assured biological safety in the case of long fibers. However, mechanical anisotropy occurs when the composites are fabricated using long fibers as reinforcements [7]. In the oral cavity, occlusal forces are applied to the teeth from various directions [8]. If the restorations are fabricated using mechanically anisotropic materials, the possibility of fracture increases when the loading is applied in a specific direction, which is problematic. Additionally, the layers of sheet fiber-reinforced materials may delaminate because of the difference in thermal expansion coefficients between the layers [7]. Therefore, attempts have been made to use short glass fibers instead of long glass fibers as reinforcing materials for dental polymers and cements [9,10]. Using short fibers solves the problem of mechanical anisotropy that occurs when long fibers are used as reinforcement. It also reduces the risk of agglomerating fillers and the possibility of air bubble contamination in the matrix resin, compared to spherical fillers. However, composite resins made from short glass fibers and dental polymers have been applied only for abutment construction after endodontic treatment or filling cavities, although they have not been used to fabricate crown restorations. Its long-term durability has not been evaluated [11,12]. Furthermore, one issue with glass-fiber-reinforced resins is that, over time, water molecules can permeate the matrix resin, causing hydrolysis between the matrix resin and glass fibers. It can result in the degradation of the material’s mechanical properties [13,14,15].

Therefore, we focused on silicon carbide fibers (SiC fibers), which are used in the aerospace industry and have superior mechanical properties and thermal resistance compared to glass fibers [16,17,18]. We hypothesized that the use of SiC fibers as a reinforcing material for dental polymers would enable the development of SiC fiber-reinforced resins with superior mechanical strength and chemical stability compared with existing fiber-reinforced dental polymers, such as glass-fiber-reinforced composites.

Chemical adhesion between the matrix resin and reinforcing materials is important to improve mechanical properties and the long-term stability of composites like fiber-reinforced resin [19,20]. Some SiC fibers contain approximately 10 wt% oxygen [16], and Si-OH can exist on the surface of the fibers. If Si-OH exists on the fiber’s surface, silanization for SiC fibers could be effective for adhering to the matrix resin and the fibers. Then, we evaluated the effect of silanization on the experimental silane coupling agent containing 3-(methacryloyloxy) propyl trimethoxy silane (γ-MPTS) for SiC fibers on the flexural properties of an experimental SiC fiber-reinforced resin. Gamma-MPTS is widely used for silanizing materials that contain silica [19,20]. From the results of our previous study, it was suggested that silanization for SiC fibers improved the flexural strength and modulus of the SiC fiber-reinforced resin. Furthermore, the SiC fiber-reinforced resin prepared in the study showed greater flexural strength than the commercial composite resins used in dentistry [21].

Then, we aimed to develop a short SiC fiber-reinforced resin with excellent mechanical properties, chemical stability, and mechanical isotropy as an alternative to continuous glass-fiber-reinforced resin using short SiC fibers as a reinforcement material.

The appropriate fiber length needs to be determined since critical fiber length exists in short fiber-reinforced composites [22,23]. In this study, the optimum SiC fiber length is determined by measuring the three-point bending strengths and bending moduli of short SiC fiber-reinforced resins containing SiC fibers of various lengths.

## 2. Materials and Methods

The materials used in the experiments are shown in Table 1.

### 2.1. Preparation of Short SiC Fibers and the Matrix Resin

Since the SiC fibers prepared for this study contain 10 wt% oxygen, Si-OH will be on the surface of the fibers. Therefore, the silane coupling treatment is applied to adhere the SiC fibers to the matrix resin. The SiC fibers approximately have a diameter of 10 μm and are bundled into 500 fibers using polyvinyl alcohol (PVA) as a sizing material. The PVA on the SiC fiber’s surface would inhibit the silane coupling reaction between the SiC fiber and the silane coupling agent. Therefore, to remove the PVA, hot water at about 100 °C was poured over the SiC fibers. The moisture that remained on the SiC fiber was then wiped off with a paper towel and air-dried at room temperature (21 °C) for approximately 1 day. Then, the dried SiC fibers were cut to dimensions of 0.5, 1, 2, and 3 mm in length with a surgical knife and used as short SiC fibers.

The short SiC fibers were placed into a polypropylene beaker, and the silane coupling agent was added dropwise and dried at room temperature while stirring to silanize the SiC fibers.

Matrix resins were prepared using UDMA, TEGDMA, CQ, and DMAEMA, as reported by Aoyagi et al. [24]. The preparation was conducted in a brown bottle within a dimly lit room. This mixture was prepared by combining 70% UDMA and 30% TEGDMA by mass. To this mixture, 0.50% CQ and 1.00% DMAEMA were added by mass and thoroughly mixed. The mixture was used as the matrix resin after the air bubbles generated during the mixing disappeared.

Short SiC fibers were mixed with the matrix resin using an automatic mixer for alginate impression materials (MIKISAN 3, YOSHIDA, Tokyo, Japan) to obtain a fiber content of approximately 6.12 wt%. Then, a short SiC fiber-reinforced resin was prepared. The fiber content was calculated based on the fiber content of the SiC continuous fiber-reinforced resin prepared in our previous study [21].

### 2.2. Preparation of Specimens

The specimen geometry for measuring the flexural strength and modulus of elasticity of the short SiC fiber-reinforced resin was 2.00 × 2.00 × 25.0 mm in accordance with ISO 4049:2009 (Dentistry—Polymer-Based Restorative Materials. ISO: Geneva, Switzerland, 2009).

**Figure 1:**
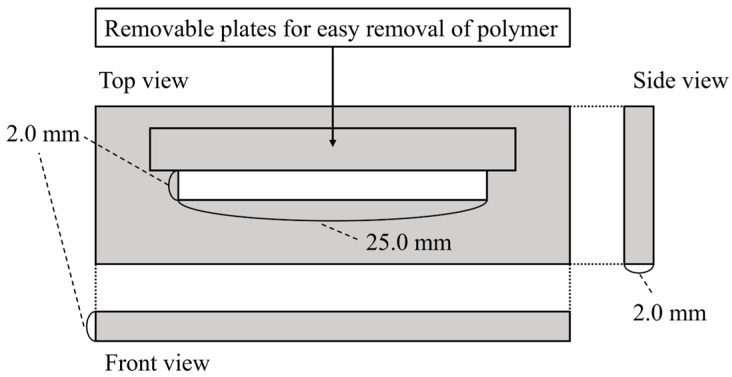
Orthographic image of the mold for preparing specimens.

A stainless-steel mold with an aperture of 2.00 × 2.00 × 25.0 mm was used for specimen preparation. A mixture of ligroin (Wako 1st Grade, FUJIFILM Wako Pure Chemical, Co., Tokyo, Japan) and paraffin wax (Paraffin wax, GC, Tokyo, Japan) in a volume ratio of approximately 1:1 was applied as a separating agent. The orthographic image of the mold is shown in Figure 1.

**Figure 2:**
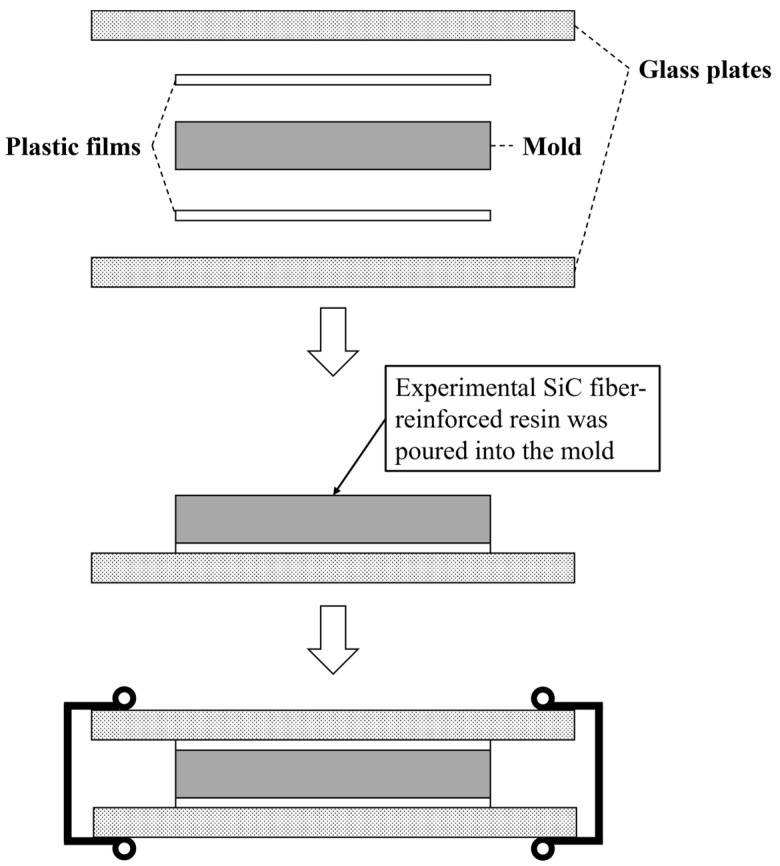
Schematic diagrams of the placement of glass plates, plastic films, and the mold. The experimental short SiC fiber-reinforced resin was poured into the mold, and the mold was combined with plastic films and glass plates.

A plastic film (Celluloid Strips, GC, Tokyo, Japan) was placed on a glass plate, and the mold was placed on top. The prepared short SiC fiber-reinforced resin was then poured into the mold so that no air bubbles formed, and a plastic film and glass plate were placed on top of the mold (Figure 2).

The glass plates were immediately irradiated with light from the top and bottom surfaces for 3 min each, for a total of 6 min, using a laboratory photopolymerizing unit that has a 465 nm–485 nm blue LED (LABOCURE HL, GC, Tokyo, Japan). After photopolymerization, the polymer and the mold were immersed in distilled water at 37 ± 1 °C for 15 min, and then the polymer was removed from the mold. The polymers were polished with #600, #900, and #1200 SiC paper under dry conditions. After that, the width and thickness of each specimen were measured using a micrometer (MDQ-30M, Mitsutoyo, Kanagawa, Japan; minimum unit: 0.001 mm). Subsequently, the polymers were immersed in distilled water at 37 ± 1 °C for 24 ± 1 h and subjected to a three-point bending test.

Table 2 lists the specimen classifications. The specimens were classified into five groups according to the presence or absence of short SiC fibers and their lengths. Each experimental group had eight specimens (*n* = 8).

### 2.3. Three-Point Bending Test

The conditions for the three-point bending test were in accordance with ISO 4049:2009, with a span length of 20.0 mm and a crosshead speed of 1.0 mm/min. The widths and thicknesses of the specimens measured after polishing were used to calculate the flexural strength and elastic modulus. The three-point bending tests were performed using a universal testing machine (Autograph AG-1000E; Shimadzu, Kyoto, Japan). The flexural strength and elastic modulus were calculated using analysis software (SHiKiBU version 1.04, Shimadzu, Kyoto, Japan).

### 2.4. Statistical Analysis

Statistical processing software (IBM SPSS Statistics, ver. 27, IBM, Chicago, IL, USA) was used to compare the means of the obtained three-point bending test results. After a one-way analysis of variance, Tukey’s HSD test was used as a post hoc test when significant differences were found (α = 0.05).

### 2.5. Fracture Surface Observation

Au-Pd sputtering was applied to the fractured specimen after the three-point bending test. Secondary electron images of the fracture surface of the specimens were captured using an electron beam microanalyzer (EPMA-1610, Shimadzu, Kyoto, Japan) to observe the fracture surface after the three-point bending test.

## 3. Results

### 3.1. Flexural Properties of the Specimens

**Figure 3:**
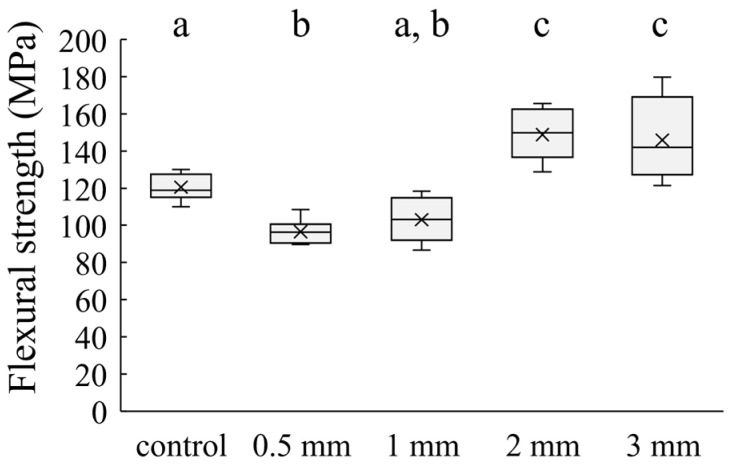
Flexural strength of each group. The bars with the same superscript were not significantly different (*p* > 0.05).

**Figure 4:**
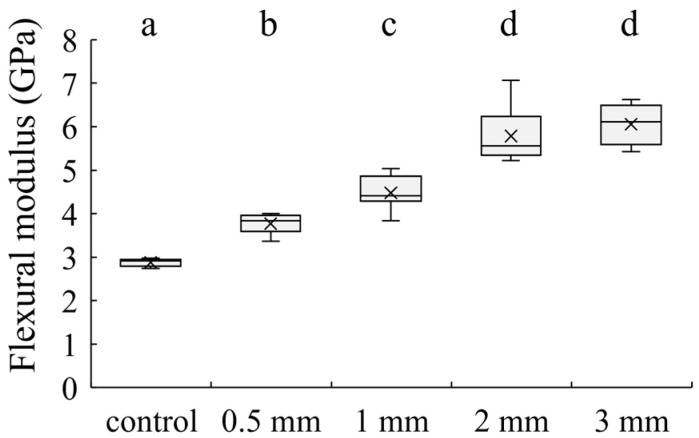
Flexural modulus of each group. The bars with the same superscript were not significantly different (*p* > 0.05).

Figure 3 and Figure 4 show the flexural strength and flexural modulus of the specimens. The mean flexural strengths of the control, 1 mm, 2 mm, and 3 mm groups were 120.5 ± 7.0, 96.6 ± 6.4, 103.0 ± 11.5, 148.8 ± 14.0, and 145.9 ± 21.8 MPa, respectively. The mean flexural moduli of them were 2.9 ± 0.1, 3.8 ± 0.2, 4.5 ± 0.4, 5.8 ± 0.6, and 6.1 ± 0.5 GPa, respectively.

### 3.2. Fracture Surface Observation

**Figure 5:**
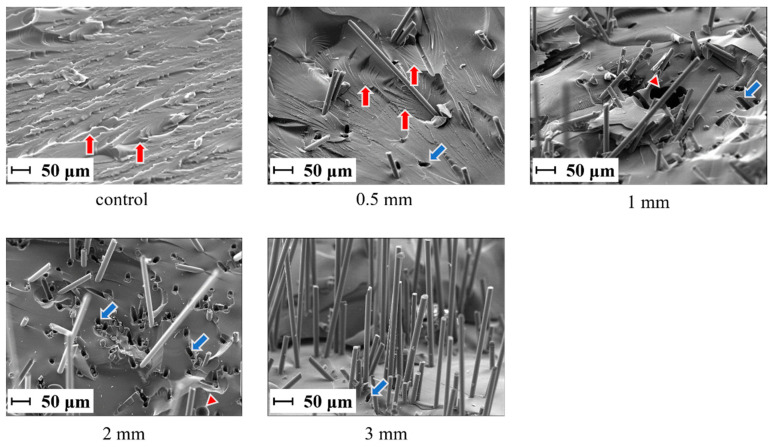
Fracture surfaces of the specimens after the three-point bending test (original magnification ×500). Red arrows indicate river patterns, blue arrows indicate holes caused by pulling out SiC fibers, and red triangles indicate bubbles inside of the matrix resin.

Figure 5 shows the results of the fracture surface observations using secondary electron images (SEI) of the specimens after the three-point bending test. On the fracture surfaces of the control group and the 0.5 mm group, a river pattern was observed. On the other hand, the fracture surfaces of the 1 mm, 2 mm, and 3 mm groups are smoother than other groups. In the fracture surfaces of the 0.5 mm, 1 mm, 2 mm, and 3 mm groups, there were holes caused by pulling out the SiC fibers.

## 4. Discussion

The three-point bending strength was lower in the groups of specimens containing 0.5 mm and 1 mm short SiC fibers than in the control groups and significantly higher in the groups of specimens containing 2 mm and 3 mm short SiC fibers. This may be related to the critical fiber length. When using fiber-reinforced materials, the fiber lengths above the critical fiber length yield major advancements in mechanical properties [22]. The critical fiber length could be calculated using the following formula: l_fc_ = σ_fu_d_f_/2τ_i_ [25], where l_fc_ is the critical fiber length, σ_fu_ is the tensile strength of the fiber, d_f_ is the fiber diameter, and τ_i_ is the interfacial shear bond strength of the fiber-resin. As for the fiber length of E-glass fiber and the mechanical properties of composites, the fiber-reinforced resins containing 1–2 mm E-glass fibers showed better fracture toughness than 8–103 μm hydroxyapatite nanofiber-reinforced resins and 20–60 μm E-glass-fiber-reinforced resins. This was attributed to the fiber length of the E-glass fibers exceeding the critical fiber length [13,26]. Although the critical fiber length of the SiC fibers used in this study may differ from the E-glass fibers because the interfacial shear bond strength (τ_i_) between the fiber and matrix resin is unknown, the results suggest that the SiC fibers increased the flexural strength of specimens when the fiber length was between 1 mm and 2 mm. These results suggest that the reinforcement effect of the SiC fibers on the flexural strength may be effective for fiber lengths between 1 mm and 2 mm.

Contrastingly, the flexural modulus was significantly higher for the specimens with short SiC fibers than for the control specimens that did not contain SiC fibers. The elastic modulus increased with increasing fiber length. Garoushi et al. reported the mechanical properties of fiber-reinforced resins when the lengths of the E-glass fibers varied from 1–6 mm. They reported that the flexural modulus of fiber-reinforced resins increased with fiber length [27]. These results suggest that the effect of the short SiC fiber length on the SiC fiber-reinforced resin was similar to the short glass-fiber-reinforced resin.

The fracture surface observations made by SEI showed no propagation of cracks between the fibers on the fracture surface of the specimens containing short SiC fibers with a fiber length of 0.5 mm. Conversely, the specimens containing short SiC fibers with lengths of 1, 2, and 3 mm showed crack propagation between the fibers; however, the presence of additional fibers effectively halted the crack propagation. For fiber lengths larger than 1.0 mm, the stress transfer between the matrix and the fibers was efficient, which suggests that the crack propagation was inhibited by the fibers.

Bubbles were observed inside the specimen, and since the bubbles and cracks were in contact with each other, they may have been the starting points for fracture transmission. Previous studies have reported that the air bubbles in composites affect crack growth, stiffness, and strength [28,29]. In this study, although the short SiC fibers and the matrix resin were mixed using a mixer for alginate impression materials that can defoam during mixing, the bubbles were mixed into the specimens. This may have caused bubbles to be introduced during the pouring of the experimental short SiC fiber-reinforced resin into the mold. Therefore, it is necessary to develop a method for preparing specimens that do not contain air bubbles, such as vacuuming the mold after filling it with resin. Abdurohman et al. reported on the comparison between the mechanical properties of glass-fiber-reinforced epoxy resin fabricated with hand lay-up, vacuum infusion, and the vacuum bagging method. According to their study, the ultimate tensile strength and elastic modulus of specimens fabricated with the vacuum infusion method were the highest [30]. Thus, the vacuum infusion method would be able to prevent the bubbles from mixing into the specimens and improve the flexural properties.

A river pattern was observed on the fracture surfaces of the control and 0.5 mm groups. The river pattern is the fracture surface observed when a brittle fracture occurs [31], which suggests that the brittle fracture occurred in the control and 0.5 mm groups. Ritchie et al. reported that the smooth fracture surfaces show no sign of ductility [32]. Therefore, the 1 mm, 2 mm, and 3 mm groups, which had smoother fracture surfaces, would have higher brittleness.

Huang et al. reported that the flexural strength and modulus of a commercial discontinuous E-glass-fiber-reinforced resin (everX Posterior, GC, Tokyo, Japan) are approximately 120 MPa and 12 GPa, respectively [33]. The experimental short SiC fiber-reinforced resin in this study had excellent flexural strength but an inferior elastic modulus to everX Posterior. The filler load of everX Posterior was 74.2 wt% for the E-glass fiber and SiO2 filler combined. However, the calculated filler load of the experimental short SiC fiber-reinforced resin was 6.12 wt%, suggesting that the difference in flexural modulus was due to the large difference in fiber content. The flexural strength of the experimental short SiC fiber-reinforced resin was higher than that of everX Posterior, despite its lower fiber content. This is due to the mechanical properties of SiC fibers, which are superior to those of E-glass fibers. In addition, the flexural strength and modulus of the S2-glass-fiber-reinforced resin fabricated by Huang et al. were approximately 190 MPa and 11 GPa, respectively. Those of the experimental short SiC fiber-reinforced resin were smaller than these values. Previous studies have suggested that the filler content may affect the flexural modulus of composites [33,34,35]. Although a similar trend was observed in this study, additional research on the interaction between the reinforcements of the short fibers and spherical fillers is needed.

On the other hand, Lassila et al. reported that the mechanical properties of composite resin containing E-glass fibers having two different length scales (200–300 μm (φ = 6 μm), so-called “microfibers”, and 1–2 mm (φ = 17 μm), so-called “millifibers”) were used as reinforcement simultaneously [36]. According to the results, the mechanical properties of the composites with fibers of two different length scales were superior to those of composites with single microfiber and millimeter fibers. Therefore, the short SiC fibers may exhibit superior mechanical strength when composited with the micrometer-scale fibers and millimeter-scale fibers used in this study.

Xu et al. evaluated the mechanical properties of specimens made with hybrid resin composites and continuous glass fiber (experimental composites) compared to specimens with fiber composite control and hybrid resin composite control. They reported that the flexural strength of the experimental composites was significantly lower than the fiber composite control, while their flexural moduli showed no significant differences. Furthermore, compared to the hybrid resin composite control, the experimental composites showed significantly higher flexural strength, work of fracture, and flexural modulus [37]. On the other hand, the flexural strength and flexural modulus of the continuous SiC fiber-reinforced resin with the same fiber content as this study were 235.0 ± 11.8 MPa and 5.0 ± 0.5 Gpa, while those of the short SiC fiber-reinforced resin prepared in this study were 148.8 ± 14.0 Mpa and 6.1 ± 0.5 Gpa, respectively. Although the continuous SiC fibers showed a superior reinforcing effect to the short SiC fibers with the same fiber content, the composite with both continuous SiC fibers and short SiC fibers would be able to exert great mechanical properties with mechanical isotropics.

## 5. Future Research Directions

The fiber content of the short SiC fiber-reinforced composite resin prepared in this study was 6.12 wt%, which is lower than the commercially available glass short fiber-reinforced composite resins, such as everX Posterior, which has a fiber content of 53.6 wt%. Since the mechanical properties of fiber-reinforced resins generally depend on the fiber content [38], it is possible to further improve the mechanical properties by increasing the fiber content of short SiC fiber-reinforced resins. On the other hand, the mixing ratio of UDMA and TEGDMA in the base resin prepared in the present study may also affect the mechanical properties of fiber-reinforced resins. H.A. Vidotti et al. reported on the effects of the fiber content of the polyacrylonitrile nanofibers and the composition of the base resin on the mechanical properties of polyacrylonitrile nanofiber-reinforced resins. There is no combination of base resin composition and nanofiber content that results in the highest flexural strength, flexural modulus, and work of fracture. Furthermore, decreases in flexural strength, flexural modulus, and work of fracture values were observed as the fiber content increased [39]. Therefore, additional studies on the effects of fiber content and base-resin composition ratio on the mechanical properties of short SiC fiber-reinforced resins would be needed.

Although UDMA and TEGDMA were prepared as monomers for the matrix resins in this study, the monomers used in dental polymer materials are not limited to UDMA and TEGDMA. I.M. Barszczewska-Rybarek reported the effects of rigid aromatic dimethacrylate (Bis-GMA), flexible aliphatic urethane dimethacrylate (UDMA), and TEGDMA on the two morphological parameters and correlated the results with the mechanical properties of polymers. The addition of UDMA to a copolymer mixture of Bis-GMA and TEGDMA increased the flexural strength, though it decreased the modulus [40]. Chih-Hsin Lin et al. reported the effects of the mixing ratio of ethoxylated bisphenol A-dimethacrylate (Bis-EMA), UDMA, and TEGDMA on the mechanical properties of copolymers. In the copolymers of Bis-EMA, UDMA, and TEGDMA, flexural strength increased significantly with increasing UDMA content, although there was no significant difference in the flexural modulus, and the surface hardness decreased. This was attributed to UDMA ester bonding [41]. Therefore, additional research about the effects of monomer type on the mechanical properties of short SiC fiber-reinforced resins would be needed.

Since SiC fibers are black in color, the light transmittance of SiC fiber-reinforced resin decreases as the fiber content increases, and photo-polymerization alone may result in a lower degree of polymerization. Ferracane et al. reported on the effects of post-cure heat treatment on fracture toughness, flexural modulus, microhardness, and the degree of conversion (FTIR). They performed post-cure heat treatments on the commercial composites at 120 °C for 10 min or 3 h immediately after light irradiation, and 24 h later, they evaluated changes in the mechanical properties. As a result, the mechanical properties significantly improved after the post-cure heat treatment after light irradiation [42]. Therefore, it would be necessary to evaluate the effectiveness of heat treatment after light irradiation, even when increasing the fiber content of SiC fiber-reinforced resin.

## 6. Conclusions

In this study, the effects of the short fiber length of SiC on the three-point bending strength and bending modulus of short SiC fiber-reinforced composite resins were investigated. The flexural strength and modulus of the experimental short SiC fiber-reinforced resin with fiber lengths of 2 mm and 3 mm were significantly higher than those with fiber lengths of 0.5 mm and 1 mm, respectively. Furthermore, the experimental short SiC fiber-reinforced resin prepared in the present study showed brittleness. The bubbles were shown on the fracture surfaces of the 1 mm and 2 mm groups. Therefore, a method to prepare specimens without any bubbles should be considered in the future.

To ensure mechanical isotropy, a short fiber length is advantageous, which suggests that a fiber length of 2 mm is appropriate for short SiC fiber-reinforced composite resins.

## Figures and Tables

**Table 1:** Materials used for preparing specimens.

Materials/Product Name	Manufacturer	Code
Urethane dimethacrylate/ Art resin SH-500B	Negami Chemical Industrial Co., Ltd., Ishikawa, Japan	UDMA
Triethylene glycol dimethacrylate/3G	SHIN-NAKAMURA CHEMICAL Co., Ltd., Wakayama, Japan	TEGDMA
Camphorquinone/ Camphorquinone	Sigma-Aldrich Co. LLC, St. Louis, MO, USA	CQ
Dimethylamino ethyl methacrylate/Dimethylamino ethyl methacrylate	Fujifilm Wako Pure Chemical Corporation, Osaka, Japan	DMAEMA
Silicon carbide fiber/ Nicalon HL-207	NGS Advanced Fibers Co., Ltd., Toyama, Japan	SiC fiber
Silane coupling agent/ Ceramic Primer II	GC Corporation, Tokyo, Japan	SCA

**Table 2:** Classification of specimens.

Groups	Short SiC Fiber	Length of Short SiC Fiber
Control	Not containing	-
0.5 mm	Containing	0.5 mm
1 mm	Containing	1 mm
2 mm	Containing	2 mm
3 mm	Containing	3 mm

## Data Availability

The data presented in this study are available upon request from the corresponding authors.
